# Endoscopic vacuum-assisted surgical closure (EVASC) of anastomotic defects after low anterior resection for rectal cancer; lessons learned

**DOI:** 10.1007/s00464-022-09274-y

**Published:** 2022-05-09

**Authors:** Kevin Talboom, Nynke G. Greijdanus, Cyriel Y. Ponsioen, Pieter J. Tanis, Wilhelmus A. Bemelman, Roel Hompes

**Affiliations:** 1 Department of Surgery, Amsterdam University Medical Centers, University of Amsterdam, Meibergdreef 9, 1105 AZ Amsterdam, The Netherlands; 2 Department of Gastroenterology, Amsterdam University Medical Centers, University of Amsterdam, Amsterdam, The Netherlands; 3 Cancer Centre Amsterdam, Amsterdam University Medical Centers, Amsterdam, The Netherlands

**Keywords:** Rectal cancer, Total mesorectal excision, Endoscopic vacuum therapy, Transanal closure, Redo-anastomosis, Anastomotic salvage, Anastomotic leakage

## Abstract

**Background:**

Endoscopic vacuum-assisted surgical closure (EVASC) is an emerging treatment for AL, and early initiation of treatment seems to be crucial. The objective of this study was to report on the efficacy of EVASC for anastomotic leakage (AL) after rectal cancer resection and determine factors for success.

**Methods:**

This retrospective cohort study included all rectal cancer patients treated with EVASC for a leaking primary anastomosis after LAR at a tertiary referral centre (July 2012—April 2020). Early initiation (**≤ **21 days) or late initiation of the EVASC protocol was compared. Primary outcomes were healed and functional anastomosis at end of follow-up.

**Results:**

Sixty-two patients were included, of whom 38 were referred. Median follow-up was 25 months (IQR 14–38). Early initiation of EVASC (**≤ **21 days) resulted in a higher rate of healed anastomosis (87% vs 59%, OR 4.43 [1.25–15.9]) and functional anastomosis (80% vs 56%, OR 3.11 [1.00–9.71]) if compared to late initiation. Median interval from AL diagnosis to initiation of EVASC was significantly shorter in the early group (11 days (IQR 6–15) vs 70 days (IQR 39–322), *p* < 0.001). A permanent end-colostomy was created in 7% and 28%, respectively (OR 0.18 [0.04–0.93]). In 17 patients with a non-defunctioned anastomosis, and AL diagnosis within 2 weeks, EVASC resulted in 100% healed and functional anastomosis.

**Conclusion:**

Early initiation of EVASC for anastomotic leakage after rectal cancer resection yields high rates of healed and functional anastomosis. EVASC showed to be progressively more successful with the implementation of highly selective diversion and early diagnosis of the leak.

**Graphical abstract:**

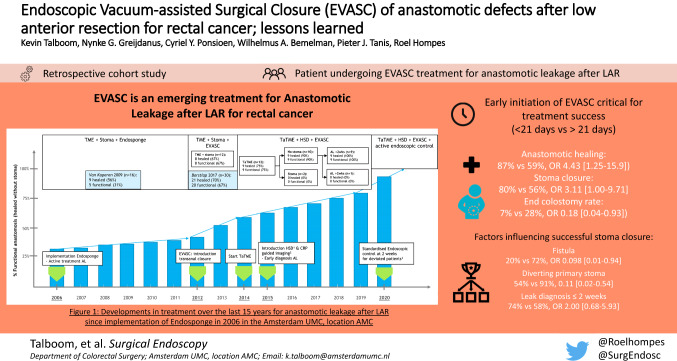

**Supplementary Information:**

The online version contains supplementary material available at 10.1007/s00464-022-09274-y.

Anastomotic leakage (AL) is still one of the most feared complications after low anterior resection (LAR) for rectal cancer, and is associated with increased morbidity, impaired functional outcomes and reduced cancer-free survival [1, 2]. Additionally, the economic burden to health care systems is high, with increased postoperative reinterventions, need for intensive care, lengthened hospital stay and readmissions [3, 4]. Despite high reported incidences of AL of up to 30% [5–7], there is very little literature on effective treatment of AL after LAR.

Conventional management of AL usually consists of faecal diversion (if not diverted primarily) and control of pelvic sepsis with transgluteal, percutaneous or transanal drainage. Rarely, dismantling of the anastomosis is required [8]. Faecal diversion and passive drainage alone do not always lead to adequate and long-term control of pelvic sepsis [9]. The internal sphincter acts as a functional barrier which causes retention, with retrograde filling of the abscess cavity behind the anastomotic defect with pus, faecal material and debris. Even if sepsis is controlled, this mechanism often prevents complete healing of the anastomosis, especially in an irradiated field [5, 9]. Failure to achieve mucosal approximation can eventually result in severe problems related to a chronic presacral sinus [10].

Endoscopic vacuum therapy (EVT) is a relatively new approach, in which vacuum-sponges are placed via the anastomotic defect into the abscess cavity [11, 12]. With negative pressure and continuous drainage, active healing of the abscess cavity is stimulated by reducing oedema, decreasing bacterial colonization and simultaneously increasing local blood perfusion that results in granulation of the perianastomotic cavity. Originally, the size of the sponge was gradually reduced during each exchange, until only a small sinus remains [13]. We adapted the technique by adding transanal closure of the anastomotic defect over a small suction drain as soon as the cavity is clean and granulating, which is named the endoscopic vacuum-assisted surgical closure (EVASC) protocol [14, 15]. This reduces the number of required sponge exchanges, aims for rapid restoration of mucosal alignment and minimizes fibrotic changes with preservation of compliance of the neorectum.

Early detection and initiation of treatment of AL is pivotal, when the neorectum is still pliable and unaffected by chronic inflammation [16]. Preliminary results from the multicentre CLEAN-study and GRECCAR group suggest that early start of EVT (< 3 weeks CLEAN and < 2 weeks GRECCAR) might increase the chance of restored continuity, but patient numbers in both studies were small [14]. This study describes the extended experience with EVASC in rectal cancer patients at the initiating centre of the CLEAN-study, with the aim to evaluate efficacy of EVASC and factors impacting success.

## Materials and methods

### Study design and patients

This is a retrospective cohort study of patients who underwent EVASC at a tertiary referral centre (Amsterdam UMC, location AMC) between July 2012 and April 2020. Patients were eligible for inclusion if aged ≥ 18 years, diagnosed with AL after TME for rectal cancer at the AMC or a referring hospital, and were managed with EVASC. Patients with a chronic sinus (a leak present > 1 year after index surgery) were excluded. Patients undergoing a redo-anastomosis were included if performed after failed EVASC, but were excluded if only preceded by a few days of EVT to clean the abscess to ensure only patients with at least one full EVASC cycle were included. The local medical ethical committee approved no written informed consent was necessary because of the retrospective nature of this study and that only a letter of no objection was sent to all eligible patients. If no objection was filed after 4 weeks, participants were included in this study.

### Diagnosis and therapeutic interventions

In our unit, TaTME was introduced at the end of 2014, and routine diversion was stopped in the beginning of 2015 with a postoperative protocol of CRP-based CT imaging of the anastomosis [17]. Referred patients were in general diverted and had conventional (either open or laparoscopic) TME surgery.

After AL diagnosis, intravenous antibiotics were started and relaparoscopy performed for ileostomy formation, if no primary ileostomy was present. In parallel, endoscopic inspection of the anastomosis and placement of the first Endo-SPONGE® (B.Braun Medical B.V., Melsungen, Germany) were carried out. If the access to the cavity was too small for the smallest insertion tube (10 mm), the leak was dilated endoscopically to facilitate the smallest calibre insertion tube of the Endo-Sponge kit. Diagnosis and initial management for AL of referred patients was according to local protocol, and EVT was started as early as possible after initial outpatient consultation at our institution.

**Fig. 1:**
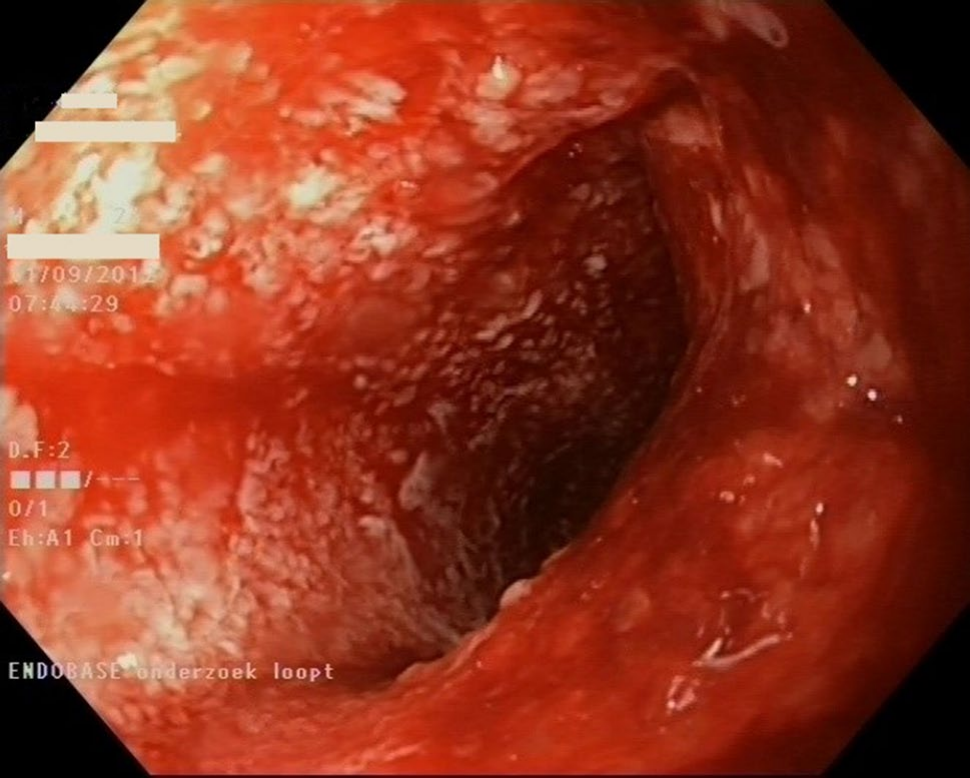
Healthy granulating tissue covering a presacral cavity after successful treatment with EVT

After initial Endo-SPONGE® placement, subsequent exchanges were performed under conscious sedation every 3 to 4 days, in an outpatient setting if possible. One or more sponges were placed depending on the size of the abscess cavity. After placement, the sponge was connected to a vacuum bottle with constant negative pressure (*Redyrob® TRANS PLUS suction device, Melsungen, Germany).* The anastomotic defect was closed surgically once sepsis was controlled and the abscess cavity was clean, showing healthy granulation tissue (Fig. 1). Details of the technique were described earlier [12, 14] and a video vignette is available online [15]. After TaTME, mostly the use of the Lonestar retractor sufficed to expose the leaking anastomosis and close the defect, while the TAMIS platform was used to close the defect for higher anastomoses after conventional TME. A drain was placed perianastomotic in the cavity through the rectal wall just below the defect. It was placed during the transanal closure procedure to ensure collapse of the presacral cavity after the procedure by negative pressure from the drain and was removed after 5–7 days [15].

Integrity of the anastomosis was checked two weeks after surgical closure by endoscopy followed by CT scan with rectal contrast. In case of failed EVASC (persisting leak), repeat EVASC was attempted if considered potentially successful. In case of persisting leakage, redo-anastomosis was offered to patients highly motivated for preserving bowel continuity. Alternatively, intersphincteric resection of the anastomosis, omentoplasty and end-colostomy was performed to treat the chronic sinus.

### Outcomes and data collection

Baseline, preoperative, intraoperative and postoperative data of the index surgery were collected until end of follow-up from electronic records and by contacting the referring hospitals to optimize completeness. Main outcome parameters were the proportions of healed and functional anastomosis at end of follow-up or at time of death. Secondary outcomes included, total number of EVT cycles and sponge exchanges, number of transanal closure attempts, anastomotic redo surgery, type of healed or functional anastomosis (primary or redo), and end-colostomy rate at end of follow-up.

### Definitions

A healed anastomosis was defined as no contrast extravasation visible on CT scan and/or an intact anastomosis during endoscopy, independent of the presence of a diverting stoma. A functional anastomosis was defined as a healed anastomosis with restored bowel continuity.

An EVT cycle was calculated from (re)start of EVT treatment until any other reintervention, such as transanal closure, or period of observation. Individual number of sponge exchanges were also calculated separately.

### Patient groups

Patients were subdivided based on the time to initiation of the EVASC protocol (date of first intervention): within 21 days of the index surgery, or later than 21 days, based on the results of the CLEAN-study [14]. Subgroup analysis was performed for (1) patients that underwent TaTME; (2) Patients with an anastomotic fistula towards the vagina, bladder or perineum; (3) patients who received a diverting stoma during index surgery; (4) Patients with leak diagnosis within 2 weeks after primary surgery and (5) Referred patients with index operation elsewhere.

### Statistical analysis

Data were either presented as mean with standard deviation or median with interquartile range, depending on the distribution, which was checked by visual inspection of the frequency distribution. Categorical outcomes were analysed using a Chi-square test and continuous outcomes using a student’s T test. Kruskal–Wallis test was used for non-parametrical continuous data. Significance was set at a *p* value of less than 0.05. Odds ratios with 95% confidence intervals were calculated for the primary binary outcomes (healed and functional anastomosis rates) and the end-colostomy rate. All statistical analyses were carried out with IBM SPSS statistics, version 26.0 (IBM, Corp Armonk, New York, United States of America). Results were reported adherent to the STROBE-statement [18].

## Results

**Fig. 2:**
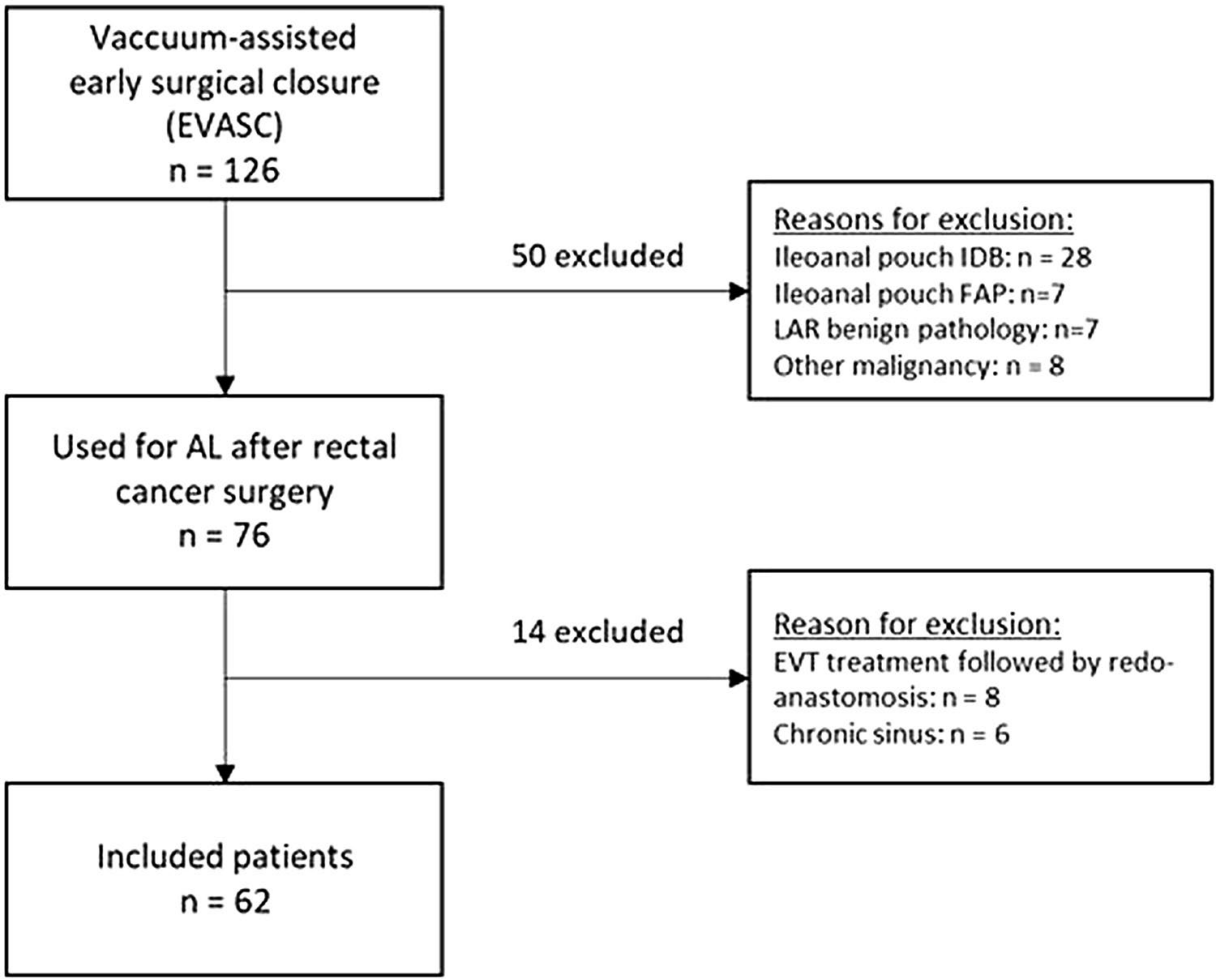
Patient flow diagram

During the study period, a total of 126 patients were treated with EVASC for leakage of a low pelvic anastomosis, of which 62 patients met the inclusion criteria and were included in the present analysis (Fig. 2). Of these 62 patients, 22 were included in the CLEAN-study [14]. Thirty-eight patients (61%) were referred after index surgery at another hospital. Patients were male in 71% and the mean BMI was 26 kg/m^2^ (Table 1). Some form of neoadjuvant radiotherapy was given in 73%. A total of 5 patients were diagnosed with an anastomotic fistula at time of AL diagnosis, which was a vaginal fistula in four patients and a fistula towards the gluteal region in one patient. Two patients had a preoperative diverting colostomy due to obstruction and 37 (61%) received a diverting ileostomy at the index operation. Median follow-up was 25 months (IQR 14–38).

**Table 1:** Baseline characteristics

	Total (*n* = 62)	LAR to EVASC ≤ 21 days (*n* = 30)	LAR to EVASC > 21 days (*n* = 32)	*p* value
Gender (male), *n* (%)	44 (71%)	22 (73%)	22 (69%)	0.691
Age in years	61 ± 9	52 ± 10	60 ± 9	0.460
BMI (kg/m^2^)	26 ± 4	26 ± 3	26 ± 4	0.738
Current smokers, *n* (%)	9 (15%)	5 (17%)	4 (13%)	0.642
ASA				
ASA 1	23 (37%)	9 (30%)	14 (44%)	0.406
ASA 2	36 (58%)	20 (67%)	16 (50%)	
ASA 3 or higher	3 (5%)	1 (3%)	2 (6%)	
Neoadjuvant therapy, *n* (%)				
SCRT	22 (36%)	5 (17%)	17 (53%)	0.003
Chemoradiotherapy	23 (37%)	10 (33%)	13 (41%)	0.553
Location of index operation, *n* (%)				
AMC	24 (39%)	19 (63%)	5 (16%)	0.000
Elsewhere	38 (100%)	11 (37%)	27 (84%)	
Surgical technique, *n* (%)				
TaTME	12 (19%)	10 (33%)	2 (6%)	0.007
Conventional TME	50 (81%)	20 (67%)	30 (94%)	
Type of anastomosis, *n* (%)				
Stapled	58 (94%)	27 (90%)	31 (97%)	0.271
Hand-sewn	4 (7%)	3 (10%)	1 (3%)	
Diverting stoma after LAR, *n* (%)				
None	23 (37%)	16 (53%)	7 (22%)	0.034
Ileostomy	37 (61%)	13 (43%)	24 (75%)	
Pre-existing colostomy	2 (2%)	1 (3%)	1 (3%)	

*LAR* low anterior resection, *EVASC* endoscopic vacuum-assisted surgical closure, *BMI* body mass index, *ASA* American Society Anesthesiology, *SCRT* short course radiotherapy, *AMC* Amsterdam Medical Centre, *TaTME* transanal total mesorectal excision, *TME* total mesorectal excision

### EVASC and other reinterventions

The EVASC protocol was started early (≤ 21 days) in 30 patients and late (> 21 days) in 32 patients after the index operation. Median interval from TME to start of the EVASC protocol was shortest in the early group (11 vs. 70 days). Interventions for AL are summarized in Table 2. Median number of sponge exchanges until transanal closure was similar in both groups (4 vs 4). Median number of EVT cycles appeared lower in the early group (1), compared to the late group (2), although not statistically significant. The majority of patients (77%) underwent only one attempt of transanal closure of the anastomotic defect.

**Table 2:** Details of EVASC and surgical interventions for anastomotic leakage

	Total (*n* = 62)	LAR to EVASC ≤ 21 days (*n* = 30)	LAR to EVASC > 21 days (*n* = 32)	*p* value
Median interval from TME to AL in days (IQR)	13 (5–28)	7 (4–13)	27 (14–46)	0.000
Median interval from TME to first reintervention^a^ in days (IQR)	17 (8–43)	9 (4–14)	42 (24 -77)	0.000
Median interval from TME to start EVASC in days (IQR)	23 (11–78)	11 (6–15)	70 (39–322)	0.000
EVT				
Median Endosponge exchanges (IQR)	4 (2–10)	4 (2–11)	4 (2–10)	0.831
Median cycles of EVT^b^, (IQR)	1 (1–2)	1 (1–2)	2 (1–2)	0.052
1 cycle	37 (60%)	22 (73%)	15 (47%)	
2 cycles	16 (26%)	5 (17%)	11 (34%)	
3 cycles	6 (10%)	1 (3%)	5 (16%)	
4 cycles	3 (5%)	2 (7%)	1 (3%)	
Retractor system used for transanal closure, *n* (%)				
Lonestar retractor	34 (55%)	18 (60%)	16 (50%)	0.429
Transanal platform	28 (45%)	12 (40%)	16 (50%)	
Median no. of transanal closure procedures (IQR)	1 (1–1)	1 (1–1)	1 (1–1)	0.832
One	48 (77%)	23 (77%)	25 (78%)	
Two	11 (18%)	5 (17%)	6 (19%)	
Three	2 (3%)	1 (3%)	1 (3%)	
Four	1 (2%)	1 (3%)	–	
Redo-anastomosis after failed EVASC treatment, *n* (%)	11 (18%)	4 (13%)	7 (22%)	0.379
Median FU in months (IQR)	25 (14–38)	22 (13–50)	27 (18–37)	0.190
Died during FU, *n* (%)	11 (18%)	8 (27%)	3 (9%)	0.075
Recurrence/metastatic disease	8 (13%)	7 (23%)	1 (3%)	0.072
Other/unknown	3 (5%)	1 (3%)	2 (6%)	

*LAR* low anterior resection, *EVASC* endoscopic vacuum-assisted surgical closure, *EVT* endoscopic vacuum therapy, *TME* total mesorectal excision, *AL* anastomotic leakage, *IQR* interquartile range

^a^reintervention could be stoma formation, EVT, combination of stoma and EVT or other interventions

^b^Cycle of EVT: one series is from start until stop of EVT therapy or until a surgical intervention (e.g. transanal closure)

### Surgical outcomes

Surgical outcomes are summarized in Table 3. Anastomotic healing rate was 73% in the total cohort, which was higher in the early group (87%), compared to the late group (59%, OR 4.43 [1.25–15.9]). The proportion of patients with a functional anastomosis at time of death or end of follow-up was also highest in the early group (80% vs 56%, OR 3.11 [1.00–9.71]). Intersphincteric resection of the anastomosis with creation of end-colostomy was performed in 11 patients (18%) of which 2 patients in the early group compared to 9 in the late group (OR 0.18 [0.04–0.93]). A redo-procedure of the anastomosis after at least one EVASC treatment was performed in 11 patients (18%), which occurred most frequent in the late group (7 patients (22%)). Causes for non-continuity in the total cohort were metastatic disease (6%), a persisting leak (6%), anastomotic fistula (15%), local recurrence (2%), patient preference (2%) and functional complaints (2%).

**Table 3:** Surgical outcomes

	Total cohort (*n* = 62)	LAR to EVASC ≤ 21 days (*n* = 30)	LAR to EVASC > 21 days (*n* = 32)	OR (95% CI)
Anastomosis healed (with or without diversion), *n* (%)	45 (73%)	26 (87%)	19 (59%)	4.43 [1.25–15.9]
Anastomosis functional (healed with restored continuity), *n* (%)	42 (68%)	24 (80%)	18 (56%)	3.11 [1.00–9.71]
Outcome related to type of anastomosis and presence of a stoma, *n* (%)				
Primary anastomosis healed, diverted	3 (5%)	2 (7%)	1 (3%)	2.21 [0.19–25.6]
Primary anastomosis healed, non-diverted (functional)	37 (60%)	21 (70%)	16 (50%)	2.33 [0.82–6.62]
Primary anastomosis non-healed, diverted	4 (7%)	2 (7%)	2 (6%)	1.07 [0.14–8.13
Redo-anastomosis healed, non-diverted (functional)	5 (8%)	3 (10%)	2 (6%)	1.66 [0.26–10.8]
Redo-anastomosis, non-healed, diverted	2 (3%)	-	2 (6%)	0.94 [0.86–1.03]
End-colostomy	11 (18%)	2 (7%)	9 (28%)	0.18 [0.04–0.93]
Reasons for non-continuity, *n* (%)				
Metastatic disease	4 (6%)	3 (10%)	1 (3%)	–
Persisting leak/chronic sinus	4 (6%)	–	4 (13%)	–
Anastomotic fistula^a^	9 (15%)	2 (7%)	7 (22%)	–
Local recurrence	1 (2%)	1 (3%)	–	–
Patient preference	1 (2%)	–	1 (3%)	–
Functional complaints	1 (2%)	–	1 (3%)	–

*LAR* low anterior resection, *EVASC* endoscopic vacuum-assisted surgical closure, *FU* follow-up, *IQR* interquartile range

^a^Including both persisting anastomotic fistula or newly developed fistula after EVASC-treatment was completed

### Subgroup analysis

Patients with an anastomotic fistula had a significantly worse healing (20% vs 77%, OR 0.074 [0.01–0.72]) and functionality rate (20% vs 72%, OR 0.098 [0.01–0.94]), compared to patients without a fistula, respectively. Patients with a primary diverted anastomosis had worse healing (62% vs 91%, OR 0.15 [0.03–0.75]) and functionality (54% vs 91%, OR 0.11 [0.02–0.54]) rates, while the end-colostomy rate (26% vs 4%, OR 7.58 [0.90–62.5]) was higher if compared to patients without a primary diverted anastomosis. Diagnosis of AL within 2 weeks showed higher healed (79% vs 63%, OR 2.25 [0.72–7.01]) and functional anastomosis rates (74% vs 58%, OR 2.00 [0.68–5.93]), although not significant (Table 4). No differences were found between patients who underwent index surgery at the AMC versus referred patients, or conventional TME vs TaTME (supplementary table 1). In 17 patients without anastomotic fistula, without primary diverting stoma and leak diagnosis < 2 weeks, healed and functional anastomosis rates were both 100%. Details are presented in Table 4.

**Table 4:** Surgical outcomes—subgroup analysis

Anastomotic fistula^a^	Fistula	No fistula	OR (95% CI)
	(*n* = 5)	(*n* = 57)	
Anastomosis healed (with or without diversion), *n* (%)	1 (20%)	44 (77%)	0.074 [0.01–0.72]
Anastomosis functional (healed with restored continuity), *n* (%)	1 (20%)	41 (72%)	0.098 [0.01–0.94]
End-colostomy, *n* (%)	2 (40%)	9 (16%)	3.56 [0.52–24.4]
Start EVASC ≤ 21 days, *n* (%)	2 (40%)	28 (49%)	-

Initial diverting stoma at index surgery	Stoma	No stoma	OR (95% CI)
	(*n* = 39)	(*n* = 23)	
Anastomosis healed (with or without diversion), *n* (%)	24 (62%)	21 (91%)	0.15 [0.03–0.75]
Anastomosis functional (healed with restored continuity), *n* (%)	21 (54%)	21 (91%)	0.11 [0.02–0.54]
End-colostomy, *n* (%)	10 (26%)	1 (4%)	7.58 [0.90–62.5]
Start EVASC ≤ 21 days, *n* (%)	14 (36%)	16 (70%)	-

Leak diagnosis ≤ 2 weeks	AL ≤ 2 wks	AL > 2 wks	OR (95%-CI)
	(*n* = 38)	(*n* = 24)	
Anastomosis healed (with or without diversion), *n* (%)	30 (79%)	15 (63%)	2.25 [0.72–7.01]
Anastomosis functional (healed with restored continuity), *n* (%)	28 (74%)	14 (58%)	2.00 [0.68–5.93]
End-colostomy, *n* (%)	6 (25%)	5 (13%)	2.20 [0.59–8.20]
Start EVASC ≤ 21 days, *n* (%)	30 (79%)	–	–

*EVASC* endoscopic vacuum-assisted surgical closure, *AL* anastomotic leakage

^a^Five patients initially presented with an anastomotic fistula towards the vagina (*n* = 4) or gluteal region (*n* = 1) and were treated with EVASC. Patients who developed an anastomotic fistula after completing EVASC were not included for this analysis

## Discussion

EVT of leaking low anastomoses is applied in our unit since 2006 [12]. Over time we moved away from the original EVT technique as described by Weidenhagen with tapering of the sponge during subsequent exchanges, and started to close the anastomotic defect as soon as the cavity was clean and granulating (the EVASC protocol). This retrospective cohort study analysed 62 rectal cancer patients who underwent EVASC, of whom the majority was referred from other institutions with delayed start of treatment. The overall proportion of healed anastomosis was 73%, and 68% had a functional anastomosis at end of follow-up. Early initiation of EVASC within 21 days after index surgery resulted in significantly higher proportion of healed and functional anastomosis. In a subgroup of 17 patients without primary diversion, without anastomotic fistula and leak diagnosis within 2 weeks, a functional anastomosis at end of follow-up was achieved in all patients.

**Fig. 3:**
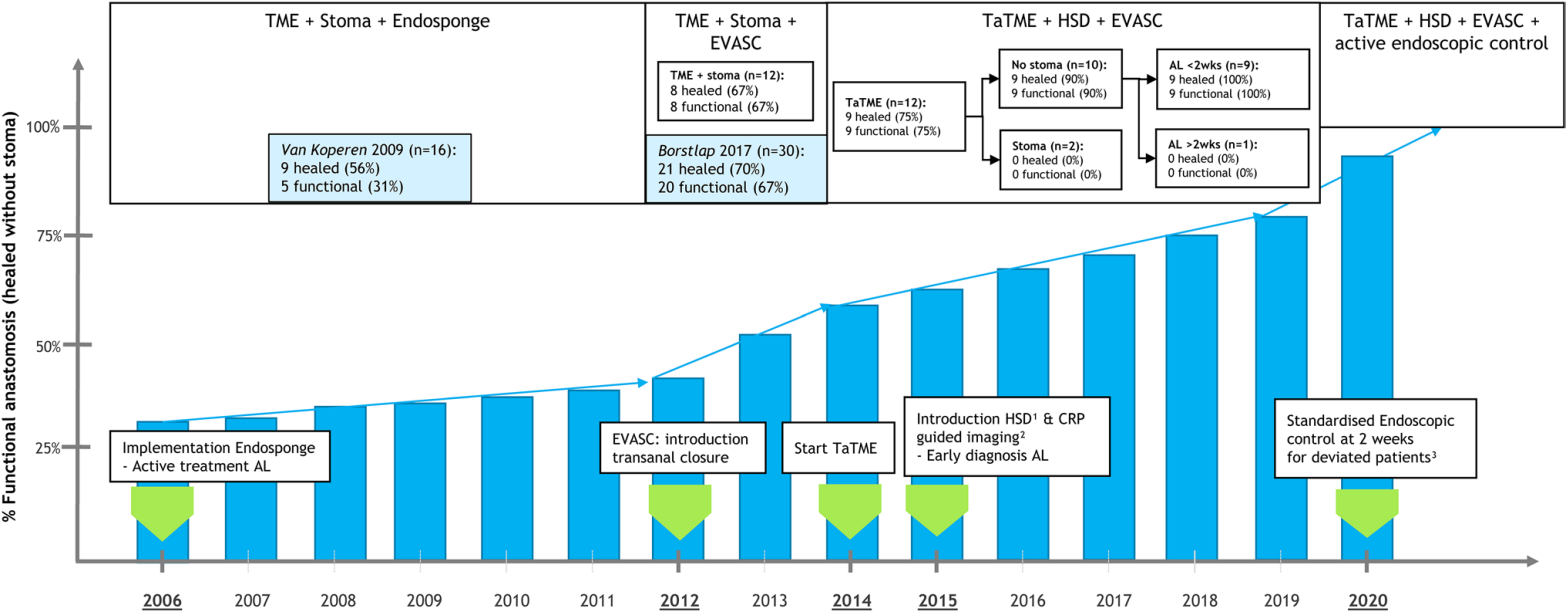
Developments in treatment over the last 15 years for anastomotic leakage after LAR since implementation of Endosponge in 2006. With implementation of HSD, a deviating stoma was only created on indication (e.g. male, obese patient with a low coloanal anastomosis). CRP-guided imaging consisted of standardised CRP-measurement on day or 4, followed by CT scan with rectal contrast when elevated. Standardised endoscopic control was not implemented in the results of this cohort. Present experience shows promising results, enabling early diagnosis despite a deviating stoma. *TME* total mesorectal excision, *EVASC* endoscopic vacuum-assisted surgical closure, *HSD* highly selective diversion, *AL* anastomotic leakage, *CRP* C-reactive protein

The increasing success rate of salvaging the leaking anastomosis reflects the evolution of the EVASC protocol. Highly selective diversion, use of a transanal platform, proactive diagnosis of anastomotic leaks using CT-guided imaging and early endoscopic assessment of anastomotic integrity increased the healing and functionality rates of the leaking anastomoses to 100% in the most recent patients. The developments in treatment over the last 15 years since the implementation of Endosponge treatment in 2006 can be seen in Fig. 3.

A recent review based on 17 studies, which included 276 patients treated with EVT for AL, found an anastomotic healing rate of 85.3% [19]. However, this review might be difficult to compare with the present study given the heterogeneity in treatment (e.g. EVT with or without transanal closure) and indication for primary surgery (e.g. rectal cancer or IBD). The GRECCAR group reported on a multicentre experience of EVT without transanal closure in 62 patients treated between 2012 and 2017 [16]. Despite exclusion of patients with an anastomotic fistula, a lower functional anastomosis rate of 55% was found after median 37 months of follow-up if compared to the present study. Similarly, they showed a higher restored continuity rate when EVT was started within 15 days (72.4% vs 27.8%). These data are in line with published results of EVT without transanal closure from our group, revealing anastomotic healing rates of 75% versus 38% using a 6 weeks cut-off [12]. However, another retrospective cohort study compared early start (≤ 21 days after LAR) with late start (> 21 days after LAR) of EVT in a small cohort of 20 patients, and found an identical anastomotic healing rate of 70% in both groups [20].

Passive treatment with local drainage and faecal diversion is often insufficient as presented in data from the Dutch SNAPSHOT collaboration [5]. One year after LAR, conservative treatment for AL resulted in a chronic sinus in half of all patients with AL. Transanal or radiological drainage of the pelvic abscess to treat AL was described in a retrospective study in 54 patients with AL after rectal cancer surgery [9]. Continuity was restored in 50% after drainage alone, and if drainage failed, a redo-anastomosis was performed in 21 patients (39%). Continuity was restored in 80% at end of follow-up and 20% had received an end-colostomy. Although many patients had their continuity restored, major salvage surgery was required more often and many lost their initial anastomosis.

Early initiation of the vacuum therapy is crucial to avoid fibrotic scarring and retraction of the anastomotic edges. Significant retraction and fibrosis of the anastomotic defect makes surgical closure technically difficult, reducing the success rate of the technique. The ability to close the defect can be assessed during each subsequent sponge exchanges. After removal of the sponge, slight suction with the endoscope will make the neorectum collapse. This enables judgement whether the two anastomotic edges reach sufficiently to make surgical closure technically possible. If the anastomotic edges are scarred and fibrotic as in late diagnosed and chronic leaks, the edges remain separated during endoscopic suction.

Early diagnosis of the leak depends on a proactive assessment of anastomotic healing using CRP-guided imaging in the non-diverted patients, and early endoscopic assessment in the diverted patients within 10–14 days after the index operation. In more recent years, we have been able to generally start the EVASC protocol within 5 days in the non-diverted patients, resulting in a high success rate.

The second factor for technical success of the EVASC protocol depends on the ease of surgical closure and therefore level of the anastomosis. Low colorectal and coloanal anastomosis done via the TaTME technique are relatively easy to close, mostly using only a Lonestar retractor.

Based on our experience, anastomotic defects can be classified according to the size of the leak and the extent of retraction present (Supplementary table 2). Significant retraction precludes surgical closure. Large defects with significant retraction or complete dehiscence due to necrosis of the afferent loop are not suitable for EVASC. The EVT can then be used for optimal sepsis control and cleansing as preparation for (an early) redo of the complete anastomosis (Supplementary Fig. 1).

Patients often received neoadjuvant radiotherapy in our cohort (73%). Radiotherapy is a known risk factor for AL and impairs wound healing due to fibrosis and reduced oxygenation of the surgical field [21]. Neoadjuvant chemoradiotherapy is also associated with larger abscess cavities, longer duration of EVT, more sponge exchanges and longer time to closure of the leak [22].

In the referred group, a longer interval to AL diagnosis, first intervention and start of the EVASC protocol was observed. This might be a reflection of the absence of a proactive protocol to assess the anastomotic integrity and the time-consuming referral process to a tertiary centre. Others were referred after failed attempts to salvage the anastomosis at the referral site.

The presence of an anastomotic fistula, for example to the vagina, can compromise successful EVASC treatment. One of the reasons for less successful EVASC in those patients might be related to the limited capacity of acquiring an appropriate vacuum seal. But fistulas to the vagina are difficult to treat anyway, and almost always require major salvage surgery [23].

In some patients, anastomotic redo surgery was performed after one or more failed EVASC attempts. Although not significant, more redo-procedures were performed in the late group (7 versus 4, *p* = 0.379). When a first attempt of EVASC has not been successful, one can decide to continue vacuum therapy in the way Weidenhagen described it originally, tapering the size of the sponge every exchange, thereby making the cavity gradually reduce in size until a small sinus remains.

Effective implementation of an EVASC protocol depends on two important factors. First, the Endo-Sponge® kit must be available. In a number of countries, the kit is not available (eg. the US), although there are off-label possibilities [24]. Second, EVASC requires a protocolised infrastructure in the surgical unit with a 24/7 availability of skilled personnel, operating theatre and endoscopic facilities.

This study has several limitations. First, all data were extracted retrospectively and missing data had to be requested from referring hospitals. However, all required data for analysing the primary and secondary outcomes were complete. Second, referral bias might have underestimated success rates. Besides, the fact that this is a single-centre experience limits the external validity of the study. Third, this study did not take the location and degree of anastomotic dehiscence into account, which is difficult to analyse retrospectively. These factors might influence the effectiveness of EVASC. Finally, although the current series is probably the largest in literature, the numbers are still small. Further research in larger cohorts (e.g. TENTACLE study [25]) can provide more definitive evidence on the most effective management of anastomotic leakage.

## Conclusion

This comparative cohort study reveals that initiation of EVASC within 3 weeks is important for successful restoration of bowel continuity after anastomotic leakage following rectal cancer resection. EVASC appeared to be progressively successful with the implementation of highly selective diversion and early diagnosis of the leaks within 2 weeks, resulting in a healed and functional anastomosis rate nearing 100%.

## Supplementary Information

Below is the link to the electronic supplementary material.

Supplementary file1 (DOCX 13 kb)

Supplementary file3 (JPG 40 kb)
