# Supplementary material for: Endoscopic vacuum-assisted surgical closure (EVASC) of anastomotic defects after low anterior resection for rectal cancer; lessons learned
Source: Surg Endosc. 2022 May 9;36(11):8280–9. doi: 10.1007/s00464-022-09274-y (PMC9613741; doi:10.1007/s00464-022-09274-y)
Supplement: Supplementary file 1 — Supplementary file1 (DOCX 13 kb) [file 464_2022_9274_MOESM1_ESM.docx]

Supplementary table 1: Surgical outcomes - subgroup analysis for AMC vs referred and TaTME vs. conventional TME

|  |  | **AMC**  **(n=24)** | **Referred (n=38)** | **p-value** |
| --- | --- | --- | --- | --- |
| **Anastomosis healed (with or without diversion), n (%)** |  | 17 (71%) | 28 (74%) | 0.806 |
| **Anastomosis functional (healed with restored continuity), n (%)** |  | 17 (71%) | 25 (66%) | 0.679 |
| **End-colostomy, n (%)** |  | 4 (17%) | 7 (18%) | 0.860 |
|  |  | **TaTME**  **(n=12)** | **TME**  **(n=50)** | **p-value** |
| **Anastomosis healed (with or without diversion), n (%)** |  | 9 (75%) | 36 (72%) | 0.834 |
| **Anastomosis functional (healed with restored continuity), n (%)** |  | 9 (75%) | 33 (66%) | 0.549 |
| **End-colostomy, n (%)** |  | 2 (17%) | 9 (18%) | 0.914 |

AMC= Amsterdam medical center; TaTME=transanal total mesorectal excision

Supplementary table 2: Classification and treatment strategy of anastomotic leakage

| **Anastomosis** | **Treatment** |
| --- | --- |
| Defect < 1/3 + no retraction | EVASC |
| Defect > 1/3 + no retraction | Partial closure followed by EVASC |
| Defect > 1/3 + significant retraction | EVT assisted early redo anastomosis |

Supplementary figure 1: Endoscopic images showing an anastomotic defect > 1/3 of the circumference with significant retraction after TaTME. Image 1 is on first endoscopic inspection after index operation. Image 2 shows a clean granulating cavity after EVT-treatment at 10 days postoperative, after which the patient underwent a successful redo-procedure of the anastomosis.
