# Supplementary figures and images for: Endoscopic vacuum-assisted surgical closure (EVASC) of anastomotic defects after low anterior resection for rectal cancer; lessons learned
Source: Surg Endosc. 2022 May 9;36(11):8280–9. doi: 10.1007/s00464-022-09274-y (PMC9613741; doi:10.1007/s00464-022-09274-y)

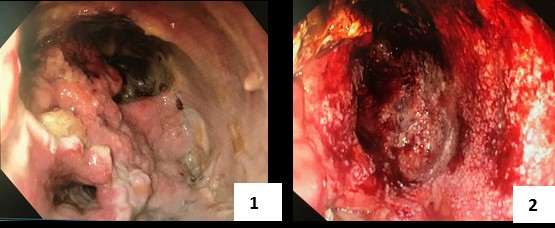

Supplement: Supplementary file 2 — Supplementary file3 (JPG 40 kb) [file 464_2022_9274_MOESM2_ESM.jpg]
